# The BETTER WISE protocol: building on existing tools to improve cancer and chronic disease prevention and screening in primary care for wellness of cancer survivors and patients – a cluster randomized controlled trial embedded in a mixed methods design

**DOI:** 10.1186/s12885-018-4839-y

**Published:** 2018-09-26

**Authors:** Donna Patricia Manca, Carolina Fernandes, Eva Grunfeld, Kris Aubrey-Bassler, Melissa Shea-Budgell, Aisha Lofters, Denise Campbell-Scherer, Nicolette Sopcak, Mary Ann O’Brien, Christopher Meaney, Rahim Moineddin, Kerry McBrien, Ginetta Salvalaggio, Paul Krueger

**Affiliations:** 1 Department of Family Medicine, University of Alberta, 6-10 University Terrace, Edmonton, AB T6G 2T4 Canada; 2 Covenant Health, Grey Nuns Community Hospital, 1100 Youville Drive Northwest, Edmonton, AB T6L 5X8 Canada; 3 Department of Family and Community Medicine, University of Toronto, 500 University Ave, Toronto, ON M5G 1V7 Canada; 4 Ontario Institute for Cancer Research, 661 University Avenue, Suite 510, Toronto, ON M5G 0A3 Canada; 5 Discipline of Family Medicine, Memorial University of Newfoundland, 300 Prince Phillip Drive, St. John’s, NF A1B 3V6 Canada; 6 Charbonneau Cancer Institute and Department of Oncology, University of Calgary, 3280 Hospital Drive NW, Calgary, AB T2N 4Z6 Canada; 7 Departments of Family Medicine and Community Health Sciences, University of Calgary, 3280 Hospital Drive NW, Calgary, AB T2N 4Z6 Canada

**Keywords:** Cancer survivors, Chronic disease, Clinical practice guidelines, Prevention, Primary care, Screening

## Abstract

**Background:**

There is a pressing need to reduce the burden of chronic disease and improve healthcare system sustainability through improved cancer and chronic disease prevention and screening (CCDPS) in primary care. We aim to create an integrated approach that addresses the needs of the general population and the special concerns of cancer survivors. Building on previous research, we will develop, implement, and test the effectiveness of an approach that proactively targets patients to attend an individualized CCDPS intervention delivered by a Prevention Practitioner (PP). The objective is to determine if patients randomized to receive an individualized PP visit (vs standard care) have improved cancer surveillance and CCDPS outcomes. Implementation frameworks will help identify and address facilitators and barriers to the approach and inform future dissemination and uptake.

**Methods/design:**

The BETTER WISE project is a pragmatic two-arm cluster randomized controlled trial embedded in a mixed methods design, including a qualitative evaluation and an economic assessment. The intervention, informed by the expanded chronic care model and previous research, will be refined by engaging researchers, practitioners, policy makers, and patients. The BETTER WISE tool kit includes blended care pathways for cancer survivors (breast, colorectal, prostate) and CCDPS including lifestyle risk factors and screening for poverty. Patients aged 40–65, including both cancer survivors and general population patients, will be randomized at the physician level to an intervention group or to a wait-list control group. Once the intervention is completed, patients randomized to wait-list control will be invited to receive a prevention visit. The main outcome, calculated at 12-months follow-up, will be an individual patient-level summary composite index, defined as the proportion of CCDPS actions achieved relative to those for which the patient was eligible at baseline. A qualitative evaluation will capture information related to program outcome, implementation (facilitators and barriers), and sustainability. An economic assessment will examine the projected cost-benefit impact of investing in the BETTER WISE approach.

**Discussion:**

This project builds on existing work and engages end users throughout the process to develop, implement, and determine the effectiveness of a multi-faceted intervention that addresses CCDPS and cancer survivorship in primary care settings.

**Trial registration:**

ISRCTN21333761. Registered on December 19, 2016

## Background

Research suggests that primary care is the ideal setting for cancer surveillance [1–3] and cancer and chronic disease prevention and screening (CCDPS) [4–6]. Reducing risk factors and detecting cancer early through evidence-based CCDPS activities is important for continued sustainability of our healthcare system [7–9]. Unfortunately, a substantial gap remains between CCDPS recommendations and actual practice [10–13]. The Building on Existing Tools to Improve Chronic Disease Prevention and Screening in Primary Care (BETTER) trial found that, on average, nine out of 28 CCDPS impactful actions were not being applied in practice [10]. The care gap is even larger for cancer survivors [14]. Despite numerous evidence-based actions and strategies that target CCDPS in primary care and are supported by clinical practice guidelines [10, 15], there are major barriers to translating this evidence into practice. For example, applying the US Preventive Service Task Force recommendations in practice was estimated to add an additional 7.4 h to a primary care physician’s day [11]. In addition to a lack of physician time, other barriers include unclear or conflicting guidelines [12, 13] and lack of enabling systems to apply these recommendations [11]. Presently, most guidelines focus on one specific cancer, medical condition, or a single risk factor [13, 16]. However, primary care providers (PCPs) need approaches that harmonize guideline recommendations across multiple conditions [13, 17].

The BETTER pragmatic cluster randomized controlled trial (RCT) demonstrated an effective CCDPS intervention in urban primary care settings [10]. The intervention utilized harmonized guidelines to address lifestyle risks through the introduction of a new role in the practice setting, the Prevention Practitioner (PP) [10, 18]. The PPs (e.g., LPN, RN, NP, dietitian) were trained on the evidence-based BETTER tool kit and obtained enhanced skills in CCDPS. They then assessed each patient’s CCDPS status based on medical records and patient self-report and met with patients one-on-one to build tailored prevention prescriptions through shared decision-making principles and motivational interviewing.

The BETTER intervention evaluated patient level outcomes using a composite index [19] of 28 evidence-based CCDPS actions (e.g., completed mammogram, measured blood pressure). The ratio of met actions to eligible actions (M/E) was 23.1% for patients receiving no intervention, and 55.6% for patients in the patient-level PP intervention arm [10].

The BETTER WISE project will build on this research by including a screen for poverty and adding a comprehensive follow-up approach for breast, colorectal, and prostate cancer survivors. Our objective is to determine whether the intervention achieves more CCDPS actions than standard care at 12 months following the initial prevention visit. We also aim to understand, if effective, how to best implement the approach into practice.

## Methods/design

### Study design

The BETTER WISE project adopts a mixed methods approach including a pragmatic two-arm cluster randomized controlled trial, and a quantitative and qualitative evaluation of the impact and implementation of the intervention.

### Research questions

Specific research aims, and questions are:

1. To evaluate the BETTER WISE intervention consisting of a PP (a new skilled role in primary care practice for cancer survivorship and CCDPS) and the BETTER WISE tool kit:
  - Is the BETTER WISE intervention effective?
  - When used in diverse settings, does the approach improve individual patients’ cancer survivor surveillance and CCDPS targets, including reduced behavioural risks, as compared to usual care?
2. To learn about the implementation of the BETTER WISE approach:
  - Is implementation in diverse primary care settings feasible?
  - What are the facilitators and barriers to uptake of the BETTER WISE approach?
  - If effective, how can it be sustained? What modifications are required to scale and spread the approach and the PP role to diverse primary care settings?
3. What is the near-term cost-benefit impact of investing in the BETTER WISE approach?

### Patient population

We target patients in the 40 to 65 age group as most cancer surveillance and chronic disease prevention and screening activities in primary care are applicable to this group [10].

### Cluster randomized controlled trial

#### Objective

The study aim is to determine if patients aged 40–65, including cancer survivors (breast, colorectal, and/or prostate) on routine survivorship care, randomized to receive an individualized prevention visit with a PP, have improved cancer surveillance and general prevention and screening outcomes as compared to standard care in a wait-list control group 12-months after the initial prevention visit.

#### Rationale for a pragmatic randomized controlled trial (RCT) design

Though the rigor of the RCT enhances internal validity, it risks limiting the external validity (i.e., generalizability) of the approach. The pragmatic design (minimal patient selection criteria, use of existing primary care practices, and tailoring of the intervention to the setting) enhances generalizability to other patients in diverse primary care practices. As such, the study will investigate the “real world” impact of the intervention. Generalizability will be enhanced by tailoring the intervention to the variable circumstances in the practices [20, 21]. The pragmatic RCT will allow us to conclude with confidence whether the intervention works over and above secular trends, something that is not achieved by a pre-vs-post-test design [22].

#### Randomization

A two-arm cluster RCT design will be used. The unit of randomization will be the physician, with participating physicians randomized to have their patients in the intervention group or wait-list control group. The physician is defined as the “cluster” to minimize the risk of contamination in that all patients in that physician cluster will either receive the intervention or wait-list control, according to the arm to which their physician was randomly assigned. Because participating physicians will be nested in a site, and some patients within a single clinical site will receive the intervention while others will not, there remains a risk of contamination. However, we perceive this risk to be low based on our BETTER trial experience [10]. Further, if contamination does occur, it would bias estimated effects towards the null hypothesis (i.e. there is no significant difference in outcomes between intervention and wait-list control groups).

#### Setting, recruitment, and sample

**Fig. 1:**
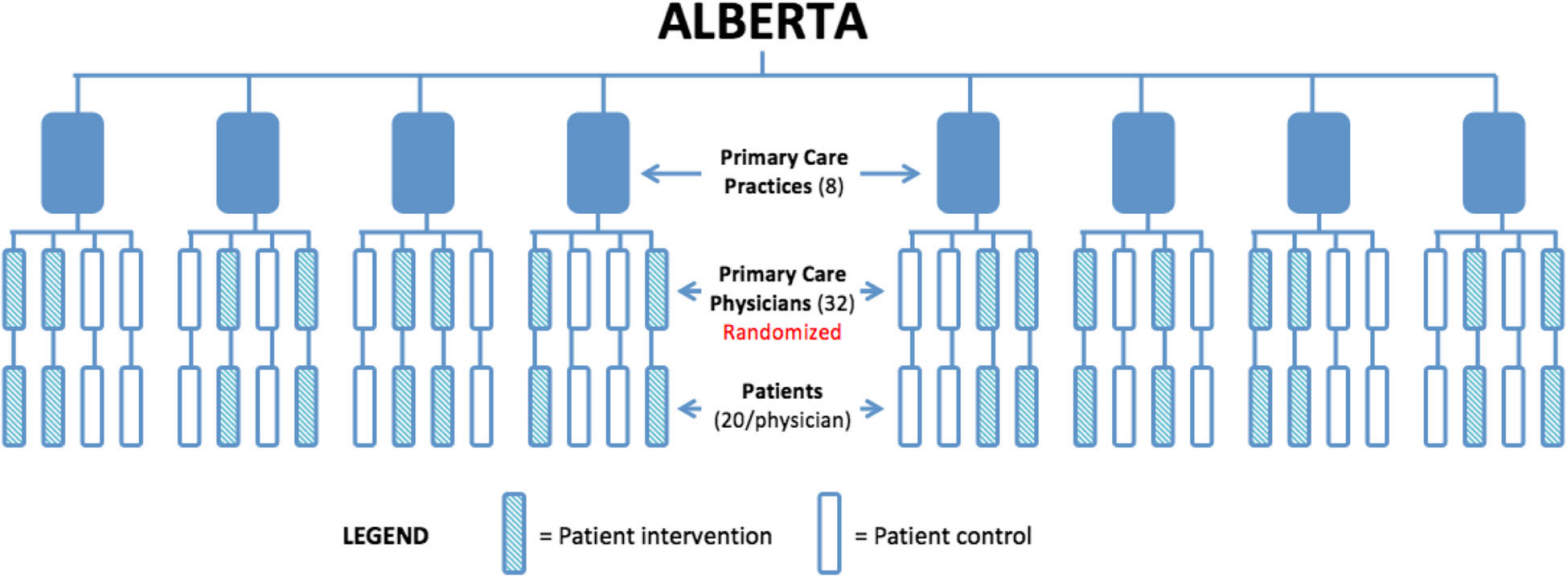
BETTER WISE Project design schema for practices in Alberta

**Fig. 2:**
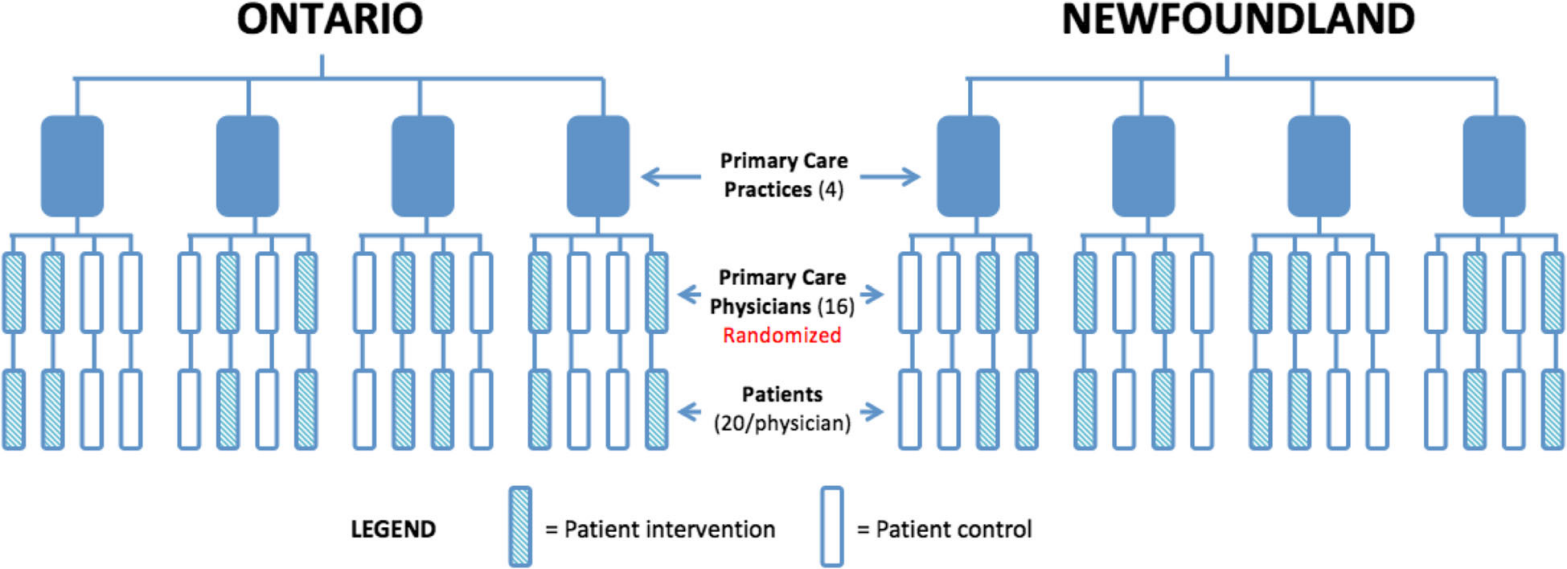
BETTER WISE Project design schema for practices in Ontario and Newfoundland & Labrador

We will collaborate with physicians, primary care groups, and health organizations in three geographically disparate Canadian provinces--Alberta, Ontario, and Newfoundland & Labrador--to identify and invite urban, rural, and remote practices to participate in our project. We will conduct this trial in 16 primary care practices and recruit 64 physicians (32 in Alberta, 16 in Ontario, 16 in Newfoundland & Labrador). This approach will result in a total of 32 physicians allocated to the intervention group and 32 physicians to the wait-list control group (see design schemas Figs.1 and 2). Each practice will identify an individual within their setting to assume the role of the PP.

All eligible breast, colorectal, and/or prostate cancer survivors will be invited; the remainder of the sample will be designated as general population patients, which will include patients with complex needs. We plan to recruit 20 patients per physician (five cancer survivors and 15 general population patients), with priority given to cancer survivors.

#### Inclusion criteria

Eligible patients include those aged 40–65 for whom we have medical record access for the previous three years. Within this sample, we will target: 1) cancer survivors (breast, colorectal, prostate) on active surveillance for recurrence (i.e. are not palliative) and 2) general population patients (i.e., who do not have a history of breast, colorectal, or prostate cancer).

#### Exclusion criteria

Patients will be excluded if: 1) they are unable to give written informed consent, 2) we are unable to access their medical history for the previous three years, or 3) they are receiving active treatment for cancer. However, patients undergoing prophylactic or hormone treatments (e.g., aromatase inhibitors) will not be excluded.

#### Identifying the cancer survivor sample

The PPs and/or primary care clinic staff working with the research team will generate a list of eligible patients for each participating physician. Charts will be reviewed to confirm, based on pathology report results, that the patient is a cancer survivor.

#### Identifying the general population patient sample

The PPs and/or primary care clinic staff working with the research team will identify eligible patients from the remaining pool. To select patients in the non-cancer survivor sample, the study biostatistician will generate a random number sequence. Patients will be invited to participate in the order of the random number sequence and assigned a unique study identification (ID) number.

Standardized invitation letters will be signed by the PP and physician. As stated in the letter, the participating primary care site will call the patient five business days after mailing the invitation to determine if they are interested in participating. If the patient meets the inclusion criteria, a verbal consent process will be completed.

#### The intervention

**Fig. 3:**
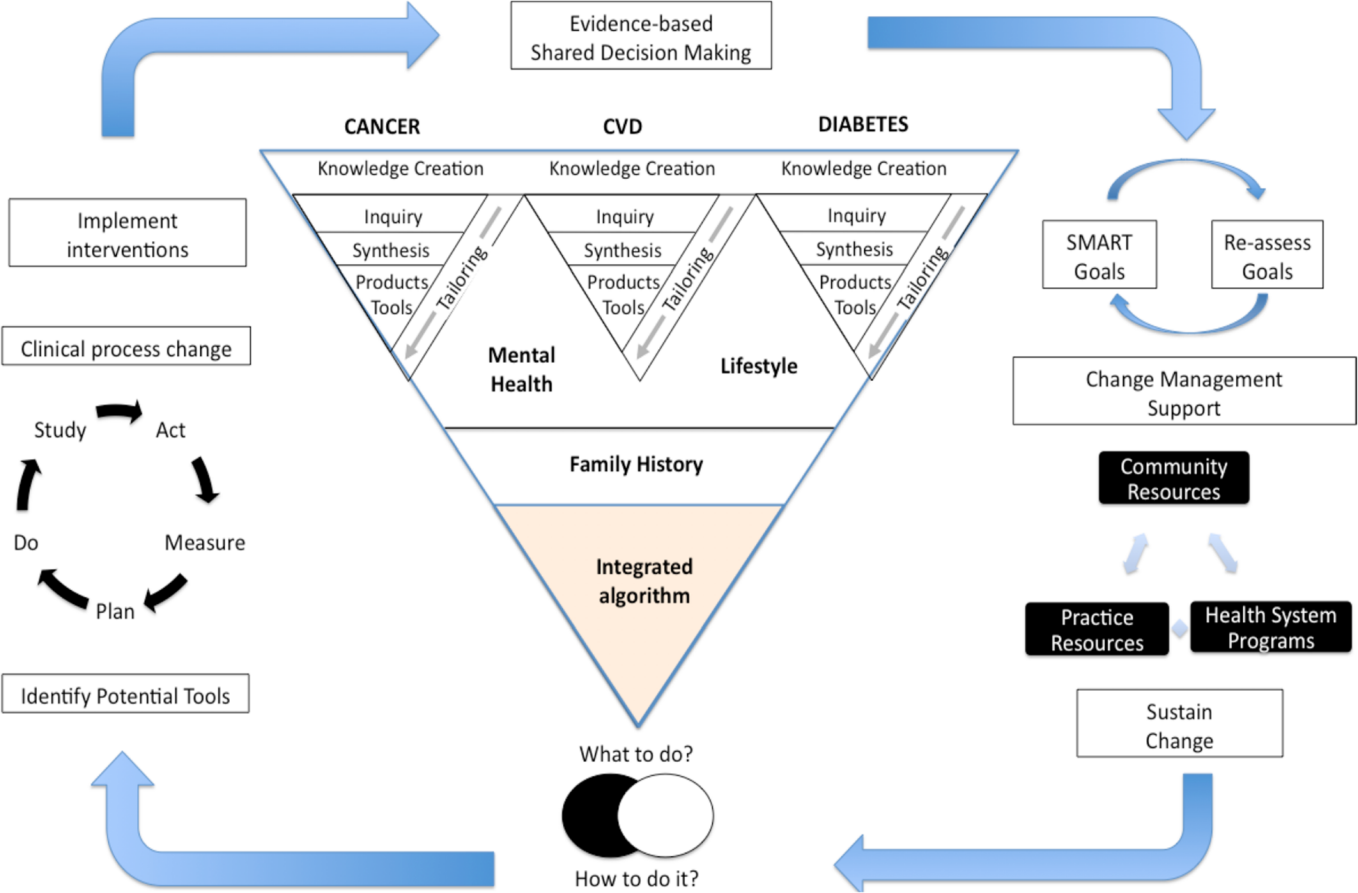
Guideline harmonization and implementation plan for the BETTER trial. The triangle in the center of Fig. 82 is an extension of the ‘knowledge creation funnel’ in the knowledge to action cycle [13, 18, 39]. The knowledge synthesis is contextually integrated for each patient. The circumference refers to the steps for implementing the tools in both the practice- and patient-level interventions of the BETTER trial. Note: CVD = cardiovascular disease, EMR = electronic medical record

The intervention consists of a one-hour prevention visit with the PP. Each practice setting will identify a clinician (e.g., LPN, RN, NP, dietitian) to take on the PP role and obtain skills in CCDPS and use of the BETTER WISE tools. The BETTER WISE tools were developed using the same process of evidence review and guideline harmonization as that used for the BETTER trial (Fig. 3) [13]. The tools received input from research, policy, and practice partners to ensure accurate and consistent CCDPS messaging. Guidelines, resources, and messages have been integrated into the BETTER WISE tool kit for CCDPS, providing a harmonized approach to prevention [8, 9, 14].

The BETTER WISE tools include: 1) the BETTER WISE health survey which includes information described in Table 1; 2) a poverty screening tool; 3) a diagram of the CCDPS targets to inform clinicians and assist with agenda setting when meeting with patients; 4) the CCDPS care maps including cancer (breast, colorectal, prostate) survivorship pathways to guide PP activities; and 5) a prevention prescription template to facilitate shared decision-making and collaborate with patients. The tools will be disseminated, published, and made available on the BETTER WISE website [23] and other websites to accelerate uptake.

**Table 1:** The BETTER health survey

Description	Data to be Collected
General health status	depression, quality of life (e.g., EQ5D), lifestyle behaviours (tobacco and alcohol use, nutrition, and physical activity [29]), including readiness for change for each of these behaviours [30]
Demographic information	sex, gender, date of birth, citizenship, ethnic/cultural background, employment status, household income, level of education, and marital status
Family history	chronic diseases including, cancers, heart disease, and diabetes
Personal history	chronic diseases, including conditions to identify patients with complex needs (e.g., hypertension, diabetes mellitus, COPD, asthma, heart failure, ischemic heart disease, chronic renal failure, mental health, obesity, addiction)
Poverty screening	

Environmental scans with input from clinicians, PCPs, and PPs will identify national, regional, and local evidence-based tools and resources to integrate into the individual PP’s BETTER WISE tool kit [24].

**Fig. 4:**
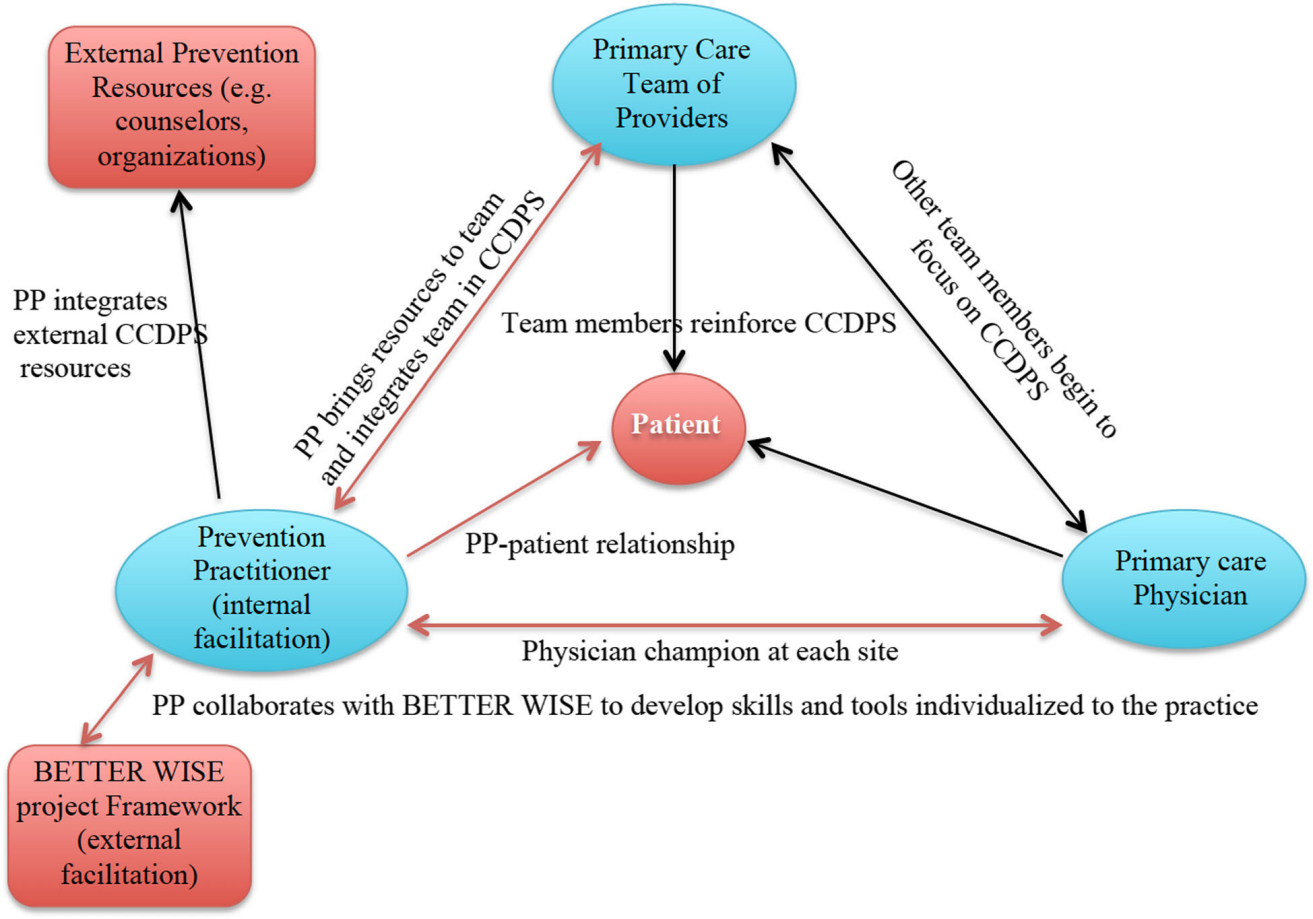
The Collaborative role of the Prevention Practitioner. CCDPS = cancer and chronic disease prevention and screening; PP = Prevention Practitioner

**Fig. 5:**
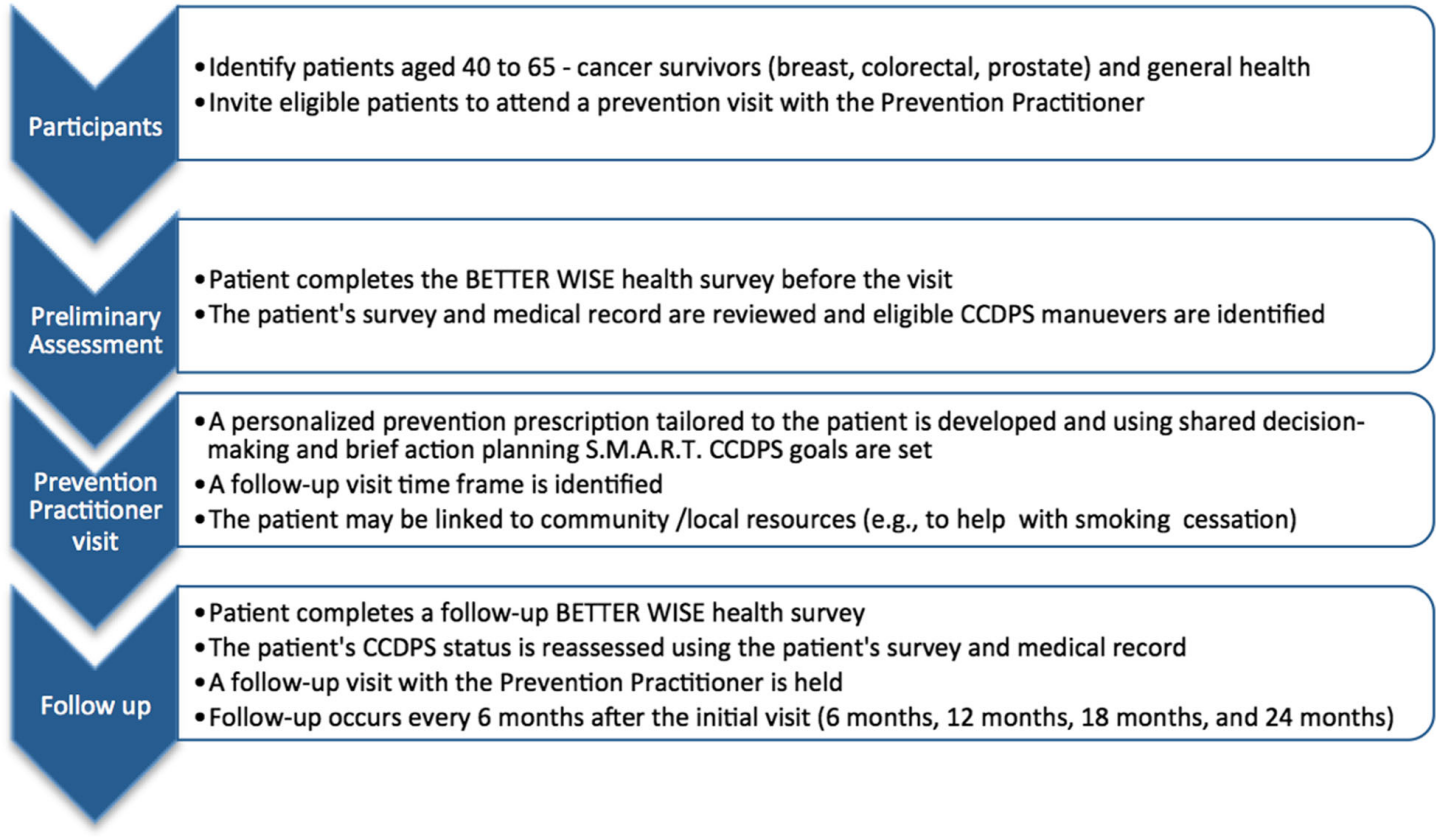
The Prevention Practitioner role in detail. CCDPS = cancer and chronic disease prevention and screening; S.M.A.R.T = specific, measurable, attainable, realistic, time-based

The PP also receives training in Brief Action Planning based on the principles and practice of motivational interviewing [25, 26] which has demonstrated feasibility and effectiveness in lifestyle interventions [27]. Clinicians participating in BETTER WISE will learn a new collaborative model of care and new roles for physicians and PPs (see Figs. 4 and 5) [18].

##### Intervention group

**Fig. 6:**
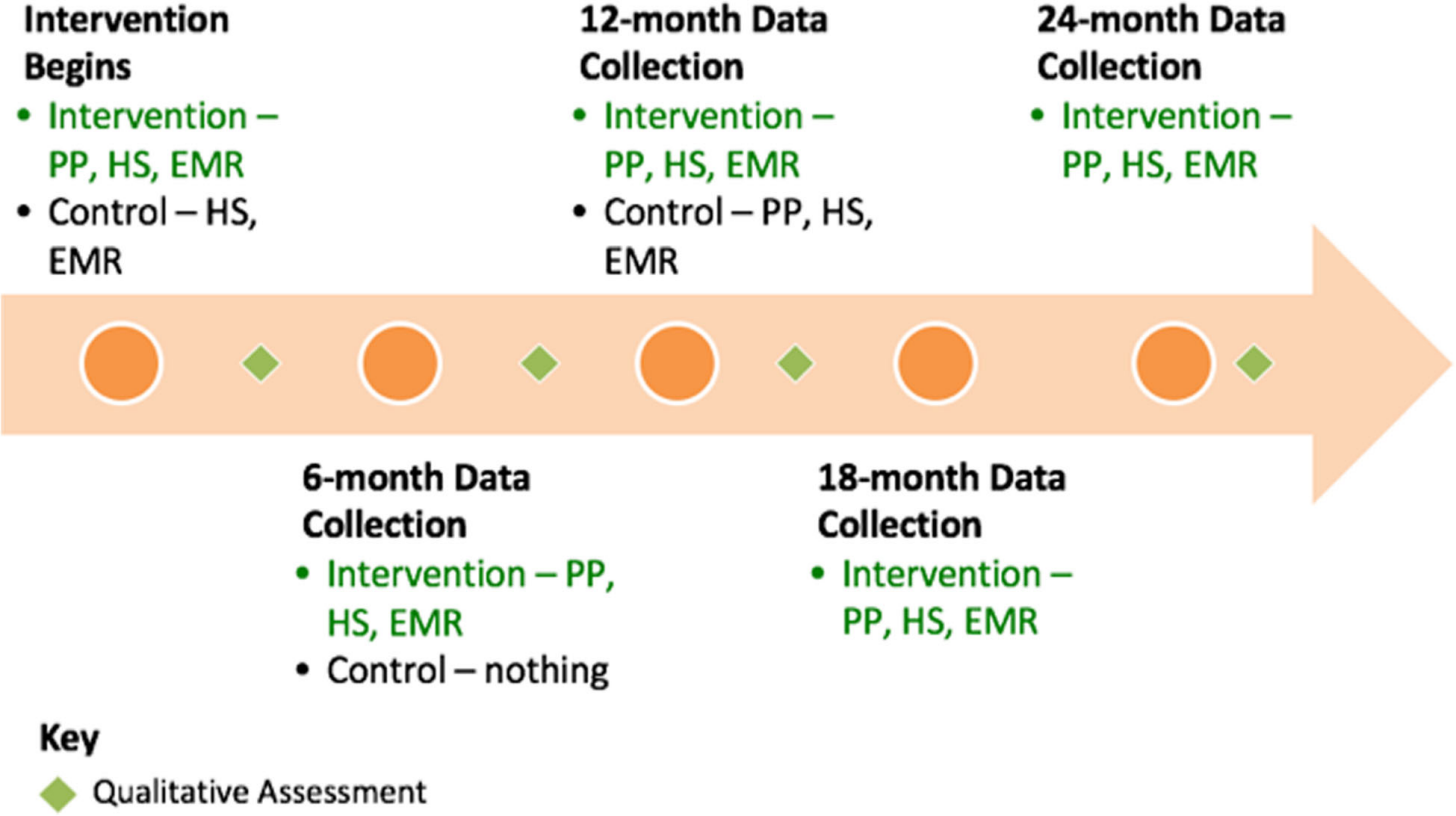
BETTER WISE Project data collection timeline. EMR = patient chart abstraction (paper or electronic); HS = BETTER WISE health survey; PP = Prevention Practitioner visit

**Fig. 7:**
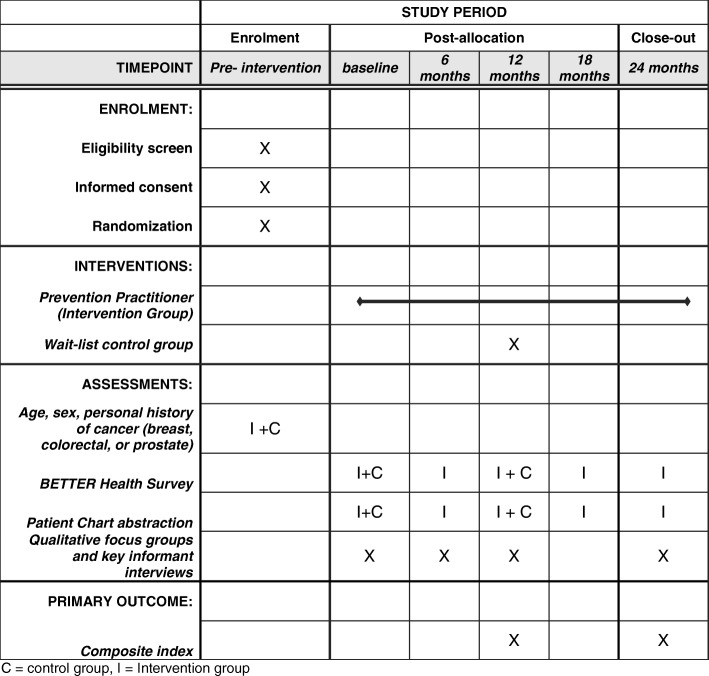
BETTER WISE Project schedule of enrolment, interventions, and assessments

Patients randomized to the intervention will be scheduled for a 60-min prevention visit with their PP. Patients will be asked to complete the BETTER WISE health survey prior to the visit and at six-month intervals up to 24 months after the initial visit (see Figs. 6 and 7). Intervention patients will also have the opportunity to review their written consent form online prior to beginning the health survey. Written consent will be obtained by the PP or another clinic staff member before the patient’s first prevention visit.

Before the visit, the PP will review the patient’s health survey responses and the medical chart to determine which prevention and screening actions the patient is eligible to receive and to prepare to discuss the patient’s risk for chronic diseases such as cancer, diabetes, and heart disease. Using shared decision-making and Brief Action Planning, the PP and the patient will develop a personalized prevention prescription. The prevention prescription, written on the standardized BETTER WISE form, may include plans for screening and referrals to programs (e.g., smoking cessation).

##### Wait-list control group

Wait-list control patients will be mailed: 1) the website link to the BETTER WISE health survey, and 2) the study consent form to completed and returned to the study team using a pre-addressed, stamped envelope. Wait-list control patients will be asked to complete the health survey immediately after agreeing to participate in the study and before their first visit with the PP, which will be scheduled upon completion of the intervention period of the study approximately 12 months later. Additional screening and prevention data will also be obtained for wait-list control patients by the PP or primary care clinic staff from the patient’s medical chart during the same data collection periods.

##### All patients

If access to a computer or the internet is identified as a potential barrier to participation, primary care sites may choose alternative methods (e.g. a paper survey) to enable patients to participate in the study.

#### Schedule of assessments and data collection

Prevention and screening data will be collected for the intervention group at six-month intervals to assess effectiveness of the intervention over 12 months and maintenance to 24 months and for secondary analysis. Each of the data collection points of the study are indicated in Figs. 6 and 7. Outcomes along with other pertinent covariate information will be retrieved from both the patient-completed BETTER WISE health surveys and patient medical records (electronic or paper).

##### The prevention and Cancer surveillance prescriptions

At the initial prevention visit, patients will be provided with a prevention prescription that summarizes their prevention and screening status, describes some goals for prevention, provides follow-up timelines, and lists any individualized screening recommendations. Prevention prescription copies will also be placed in patients’ medical records and will be used as a secondary data source. The prevention prescriptions will capture the information as outlined in Table 2.

**Table 2:** Prevention Prescription Data

Description	Data to be Collected
All relevant chronic disease prevention and screening test results and values	cholesterol, fasting blood sugar, glycated hemoglobin, stool for blood, sigmoidoscopy, colonoscopy, pap, and mammogram
All relevant cancer surveillance results and values depending on personal history of cancer	breast - mammography, bone density; colorectal – colonoscopy, carcinoembryonic; antigen test, computerized axial tomography; prostate - prostate specific antigen test
Physical and vital information	blood pressure, weight, height, and body mass index For patients randomized to the intervention group, the PP will take the BP, weight and height measurements during the visit with the patient. For patients randomized to wait-list control, these will be collected from their medical record (electronic or paper) at baseline and 12-month data collection time points.
Referrals made or actions to be taken by the patient, PP, or primary care team in order to follow up on identified CCDPS items	Description of referrals and actions taken

##### The S.M.A.R.T. goals sheet

Along with the prevention prescription, patients will receive a copy of their S.M.A.R.T (specific, measurable, attainable, realistic, time-based) goals sheet. The prevention prescription informs the patient about their CCDPS status. After reviewing the patient’s CCDPS status, the PP will use shared decision-making and Brief Action Planning to help patients make S.M.A.R.T goals for their health. These will be documented on the S.M.A.R.T. goals sheet, as will the patient’s confidence in achieving each goal. Patients will be advised to make a maximum of three goals to ensure that achievement of each is feasible. Copies of the goals sheet will also be filed in patients’ medical records and used for secondary data analysis.

### Evaluation and impact of the intervention

#### Outcomes and statistical analysis

**Fig. 8:**
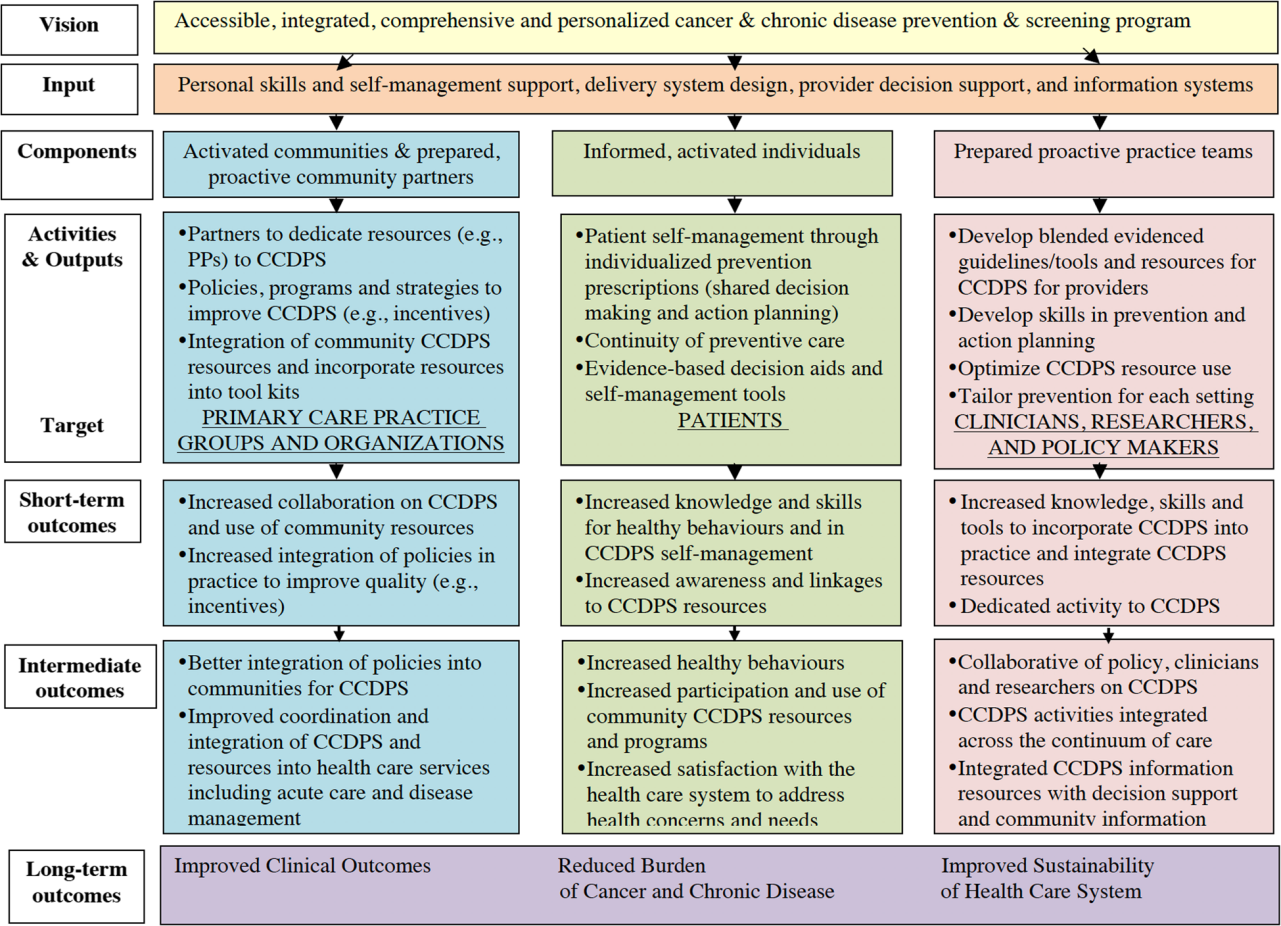
BETTER WISE Project logic model. CCDPS = cancer and chronic disease prevention and screening

**Fig. 9:**
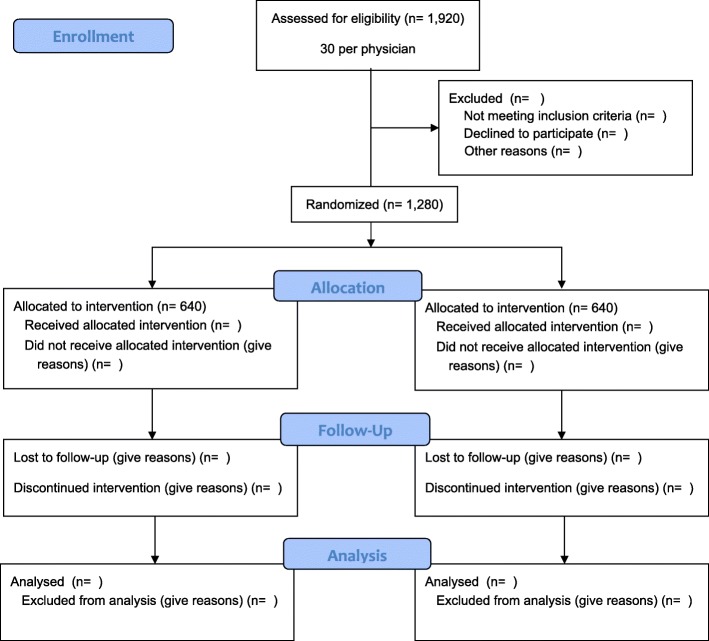
BETTER WISE Project CONSORT flow diagram

Outputs and outcomes include both practice- and patient-level outcomes (Fig. 8). To demonstrate the effectiveness of BETTER WISE, we will determine if cancer surveillance care and general prevention and screening outcomes improve for patients who receive an individualized visit with a PP, as determined by a composite index, when compared to wait-list controls at 12 months follow-up. The analysis will adhere to the intention-to-treat principle in that none of the enrolled patients will be excluded from analysis and all patients will be analyzed according to the randomization scheme (see Fig. 9). The unit of analysis will be the individual patient.

##### Sample size calculations

Based on the findings from our previous research (BETTER trial and BETTER 2 study), we estimate the intra-class correlation coefficient (ICC) to be 0.30. From the BETTER trial, we anticipate the percentage of achieved CCDPS actions in the wait-list control arm will be approximately 20%. To detect a 20% intervention effect (i.e., the proportion of achieved CCDPS actions for patients randomized to the BETTER WISE intervention is 40%, compared to 20% for wait-list control) with 80% power, we will need to recruit approximately four patients per physician to achieve our sample size requirements per area of interest (cancer survivors and general population patients) at 5% alpha. The 20% intervention effect size is conservative given information gathered from the BETTER trial. As such, we would require four cancer survivors and four general population patients for each participating physician to have sufficient power to conduct sub-group analyses (i.e. measuring the effectiveness of the PP intervention in cancer survivors and the general population patients).

We will also target patients with complex medical needs as described by the province of Alberta’s billing definition of complex needs. In the BETTER trial, 25% of patients 40 to 65 years of age met the Alberta’s billing criteria for complex needs. To guarantee that we will recruit enough patients to meet the definition of complexity, we will increase our total sample size to 16 patients (four cancer survivors and 12 general population patients) for a total of four patients with complex needs (i.e., 25% of 16 = 4). This will allow sufficient power to conduct sub-group analyses with respect to measuring the effectiveness of the PP intervention on patients with complex needs.

To account for possible study withdrawal and/or loss to follow-up, we will further inflate our sample size by 25% for a total of 20 patients per physician (five cancer survivors and 15 general population patients) for a total sample size of 1280 patients (320 cancer survivors and 960 general population patients). A total of 30 patients per physician will be recruited to participate in the project to ensure our target sample size of 20 patients per physician is met. Participating practices may choose to over-sample cancer survivors or patients who meet the definition of complex needs in order to reach their target sample (five cancer survivors and 15 general population patients).

#### Primary outcome and analysis

Effectiveness of the PP intervention will be assessed via a comparison of the composite outcome between intervention and control groups at 12-months. The primary outcome and performance indicator will be a composite index [19] of evidence-based chronic disease prevention and screening actions related to diabetes, heart disease, cancer screening, lifestyle factors associated with those chronic diseases and, for cancer patients, appropriate cancer follow-up care calculated at 12 months. The BETTER WISE composite index will be identical to the BETTER trial index in its mathematical definition, but items in the index may differ. The composite index is calculated as the total number of CCDPS actions met divided by the total number of CCDPS actions the patient is eligible to receive, multiplied by 100. The composite index is treated as a continuous outcome, expressed as a percentage that ranges from 0 to 100 and calculated at the patient level. A composite outcome will be used because it is not feasible to power the study to assess each individual item (multiple comparisons). We will revise the index content developed for CCDPS and adapted from the BETTER trial [10, 24] to reflect the CCDPS messages and actions recommended in each participating province to be consistent with jurisdiction-specific messaging.

We will examine the distribution of composite outcome for normality and will use proper transformation, if needed. Descriptive statistics (mean, standard deviation, median, quartiles for continuous variables, composite index) and counts/percentages for categorical variables (gender, level of education, etc.) will be used to describe the sample. The outcome measures and their components will be summarized at baseline and the end of the study using appropriate summary statistics for each physician, primary care practice, province, and overall. Generalized Estimation Equation (GEE) methods will be used for assessing the impact of interventions on prevention and screening of cancer and other chronic diseases in primary care. The GEE methodology provides a powerful method of analyzing correlated (clustered) data. We will estimate the intervention effect on the composite index and on the sub-components for the overall sample, and in the cancer and general population strata, respectively.

#### Secondary outcomes

Secondary outcomes will include 1) proportion of patients meeting the evidence-based targets, as measured by tracking specific CCDPS actions (e.g. mammograms completed) longitudinally at six, 12, 18 and 24 months, 2) number of targeted CCDPS actions that patients randomized to the intervention group were deemed eligible to undertake at baseline that are met (according to pre-defined targets) at 24-month follow-up, 3) a 24-month composite index score considering the number of targeted CCDPS actions patients were deemed eligible to improve at baseline, and 4) sub-analyses for the primary and secondary outcomes performed for cancer survivors and complex needs patients, where sample size permits.

#### Economic assessment

We will perform an economic evaluation of the intervention from the perspective of the health care payer, taking into account program costs and costs incurred by the recommended actions. We will estimate program costs in two ways: 1) by assuming that costs associated with the intervention are borne by the system directly and 2) by assuming that costs are absorbed via physician billings.

#### Evaluation of implementation

A qualitative evaluation will focus on the perspectives of patients, health care providers (including physicians and PPs), and other community members involved in the project. The purpose of the qualitative evaluation is to understand the impact of the approach as well as the implementation of the approach (e.g., impact on patients and practices including perceived benefits and disadvantages), and the uptake process (e.g., perceived barriers and enablers) including sustainability in different primary care contexts.

After the review of numerous implementation frameworks [24, 28–30], two frameworks were consistent with the study objectives [24, 28, 29]: 1) the ADKAR (Awareness, Desire, Knowledge, Ability and Reinforcement) [29] model will inform the qualitative evaluation on a micro level (individuals and individual practices) and 2) the CFIR (Consolidated Framework for Implementation Research) [28] model that will help us to understand implementation on a meso and macro level (i.e., overall picture, policy level). The qualitative component will complement Phase 2 by assisting with: 1) implementing our RCT in the practice setting and identifying early facilitators and barriers and 2) a deeper understanding of the practice context to help interpret our quantitative findings. Initial and follow-up focus groups and interviews of key informants (e.g., PPs, administrative staff, clinicians, allied health professionals) will be analyzed with constant-comparative methods [24]. All focus group and key informant interview participants will provide voluntary, written informed consent before participating.

Because participation outside of the prevention visit was challenging for patients in the original BETTER trial, we will invite patients to fill out a short feedback form following their appointment rather than inviting them to participate in a qualitative interview or focus group. In the previous implementation of BETTER [24], we found that a simple open-ended survey provided valuable responses from over 50% (*n* = 91/154) of patients. The survey asked three open-ended questions: 1) what patients liked about their visit, 2) what they would have liked to be different about their visit, and 3) other comments. For BETTER WISE, we expanded the patient survey by including the ASK-MI (Alberta Shared Decision Making Measurement Instrument) to gather more information about the prevention visit, specifically the level of shared decision-making between patient and PP dyads. The ASK-MI tool contains six Likert scale items and matches patients’ experiences of their prevention visit with PP’s responses of the same visit (e.g., to what extend the PP and the patient worked together to agree on a plan and the main concerns of the visit). BETTER WISE data captured by patient feedback surveys will be analyzed quantitatively using descriptive statistics and qualitatively using thematic content analysis.

#### Further assessment informed by the RE-AIM framework elements

The RE-AIM framework provides a strategy to assess programs that work in the real-world setting [31, 32]. Three specific items will be evaluated including implementation at the level of the patient.

1. Reach – the number of patients who agree to participate and who follow through with PP visits.
2. Effectiveness – measured using a composite index [10, 24] and summary statistics.
3. Adoption, Implementation and Maintenance – qualitative and quantitative methods to explore BETTER WISE feasibility, facilitators and barriers to implementation, adaptation, and maintenance over 24 months for patient care in diverse urban and rural settings.

### Project management considerations

#### Data management

Study data will be collected and managed using REDCap [33] electronic data capture tools hosted and supported by the Women and Children’s Health Research Institute at the University of Alberta. REDCap, is a secure online platform used for building and managing surveys and databases.

#### Timeline

**Fig. 10:**
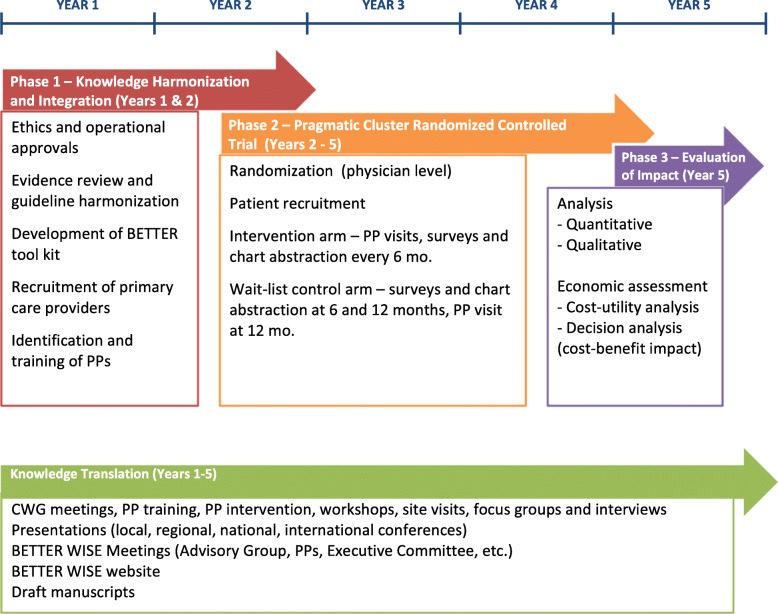
BETTER WISE Project timeline

Figure 10 outlines the funding timeline. The significant milestones for Phase 1 are: 1) evidence review and update; 2) BETTER WISE tools developed; 3) tool kit integrated and refined; 4) templates developed for electronic medical record (EMR) tool; 5) BETTER WISE electronic templates refined for use on an electronic platform; and 6) BETTER WISE training modules developed.

Phase 2 of the project includes the pragmatic two-arm cluster RCT. The significant milestones are: 1) ethics approval obtained; 2) physicians and practices recruited, physicians randomized for RCT; 3) clinicians identified to take on role of PP and trained; 4) patients recruited; 5) completed PP visits with intervention and waitlist control patients.

Phase 3 of the project is the evaluation of impact.

#### Research ethics considerations

Approvals from the relevant research ethics boards for participating sites in each of the three provinces will be obtained prior to the commencement of the trial. All patients in the research trial will provide written informed consent. Individuals who are not able to provide consent for reasons of language, literacy, or competency will be excluded. Since all elements of the patient-level intervention are consistent with best practice and could be offered as part of usual patient care by PCPs, there are no risks or harms associated with this approach.

##### Confidentiality

Individuals participating in this study will be made aware during the consent process that the research team will have access to their personal health information and will collect only information needed for this study. Personal identification will be stored securely in a locked cabinet at the corresponding PCP’s clinic for a minimum of seven years, as will study information collected from patients used to inform ongoing care (e.g. the prevention and cancer surveillance prescriptions).

The personal health information collected in this study will only consist of that information that is relevant to prevention and screening of cancer and other chronic diseases and may include: 1) medical history including medications, 2) test results (e.g., blood tests), 3) mammogram or other test reports, and 4) reports about visits their primary care provider, treatments, and side effects.

Qualified representatives from each of the participating sites, as approved by the respective research ethics boards, may also access personal health information to verify that the information collected for the study is correct, confirm the prevention prescription, or make sure that the study is adhering to all regulatory requirements. In addition, these representatives may receive health information that is collected specifically for this research (“study data”). However, to protect identity, any study data that are sent outside of the PCP’s clinic will not contain personal identifiers; rather, a unique study ID number will be captured for these documents.

#### Knowledge translation and exchange

**Fig. 11:**
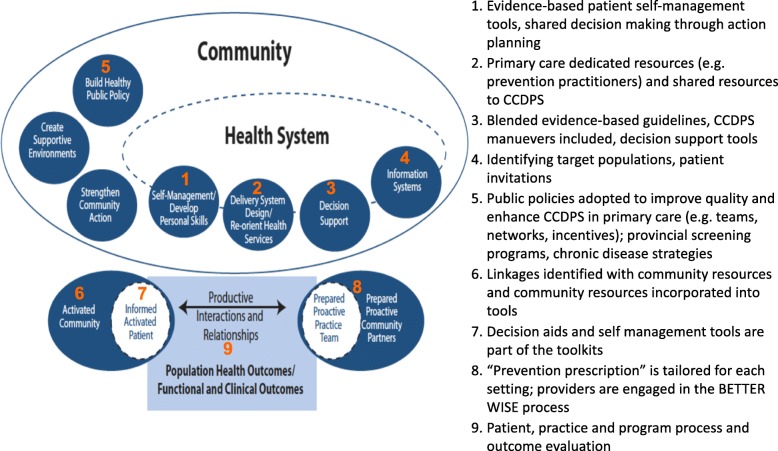
BETTER WISE Project mapped onto The Chronic Care Model. CCDPS = cancer and chronic disease prevention and screening

Our primary objective is to improve uptake of CCDPS, including cancer survivorship care (Fig. 8). BETTER WISE maps conceptually onto the Expanded Chronic Care Model [24, 34] (see Fig. 11). By bringing together practice end-users with research, policy, and decision makers in Phase 1 of our project (Fig. 3), we can develop comprehensive CCDPS resources and tools that will integrate our varied perspectives in key messages and actions to be applied in the practice setting.

We will use a collaborative approach of integrated knowledge translation [35] to engage our end-users throughout the project. Identified end-users include patients, primary care practitioners, and policy makers. Our team’s expert patient advisors and patient feedback will add patient perspectives to the research process. Collaboration among end-users and researchers throughout the research process will keep outputs relevant to and practical for end-users (Fig. 3) [35–37]. Collaboration will assist with tailoring consistent messages to specific audiences (e.g., PCPs, patient). Working with primary care practices will help us blend and tailor an approach that is actionable in the practice setting.

We aim to change models of care because physicians lack the time for CCDPS in traditional models of care [11]. Practices will designate a clinician to take on the PP role and become a resource to the practice by developing expertise in CCDPS and use of the BETTER WISE tools. We will use qualitative and descriptive methods to evaluate the BETTER WISE intervention implementation. An economic cost utility analysis will inform stakeholders of the cost and sustainability benefits to the health care system of our approach.

## Discussion

The BETTER WISE project facilitates collaboration among PCPs, clinicians, patients, researchers, policy makers, and decision makers across Canada to improve CCDPS in primary healthcare. Working together the BETTER WISE clinical working group will identify evidence-based resources and supports to help their patients improve their lifestyles, including prevention and screening for cancer and chronic diseases, as well as develop care paths for breast, colorectal, and prostate cancer survivors. The project involves engagement and integration since national, regional, local, and clinical resources and supports are being incorporated into the care paths and resources. Through the integration of these resources, the PP will be able to link patients to resources that can best help them to achieve their goals (e.g., linking patients to locally available smoking cessation programs). Healthy lifestyles, wellness, and risk modification are specifically targeted through behavioural lifestyle interventions that are informed by theories of behavioural change, which will improve the likelihood that the interventions will succeed and result in improved healthy behaviours. The patient surveys will include assessments of lifestyle risk factors (diet, obesity, physical activity, alcohol, tobacco, and lack of screening), including the patients’ readiness and confidence to change their lifestyle. Performance indicators will capture multiple domains, including changes in the practice and health behaviour and physiological changes in patients. We will identify CCDPS targets in these domains and develop a composite index to measure them.

Participating family practices will identify an individual to take on a new role (PP), becoming an expert in prevention for the practice. A BETTER WISE tool kit developed by the BETTER WISE clinical working group will facilitate the implementation of evidence into practice and guide clinicians on how best to help patients holistically achieve their health goals. We will evaluate the implementation of the PP role into practice to better understand the barriers and facilitators to implementation.

This study is important from the perspective of prevention of cancer and other chronic diseases in middle-aged adults as well as from the perspective of the health care system. For middle-aged adults, prevention programs and screening actions for chronic diseases, if effective, can prolong good health. For the health care system, this intervention must be practical, generalizable, and cost-effective. Also, understanding how to best implement a new approach into practice that includes patient level outcomes is important for future program implementation activities. With the prevalence of heart disease, diabetes, and cancer, and the competing demands on the PCP practices, the need to find better methods to facilitate the use of screening and prevention programs cannot be overstated [38].

## Abbreviations

ADKAR: Awareness, Desire, Knowledge, Ability, Reinforcement
BETTER trial: Building on Existing Tools to Improve Chronic Disease Prevention and Screening in Primary Care; a pragmatic randomized controlled trial in Alberta and Ontario
BETTER 2: Building on Existing Tools to Improve Chronic Disease Prevention and Screening in Primary Care; an implementation study in Newfoundland & Labrador and the Northwest Territories
BETTER WISE: Building on Existing Tools to Improve Cancer and Chronic Disease Prevention and Screening in Primary Care for Wellness of Cancer Survivors and Patients
CCDPS: cancer and chronic disease prevention and screening
CFIR: Consolidated Framework for Implementation Research
EMR: Electronic medical record
GEE: Generalized estimation equation
ICC: Intraclass correlation coefficient
LPN: Licenced Practical Nurse
NP: Nurse Practitioner
PCP: Primary care provider
PP: Prevention Practitioner
RCT: Randomized controlled trial
RE-AIM: Reach, Effectiveness, Adoption, Implementation, Maintenance
REDCap: Research electronic data capture
RN: Registered Nurse
S.M.A.R.T: Specific, measurable, attainable, realistic, time-based
